# The Year-Book of the Nose, Throat and Ear for 1900

**Published:** 1900-04

**Authors:** 


THE YEAR-BOOK OF THE NOSE, THROAT AND EAR FOR 1900. By Drs. J. P. Head and Albert H. Andrews, of Chicago, Published by Chicago Medical Book Co., Chicago, Ill.

This book of 274 pages has just been issued. It is a résumé of the work done, as published in the various medical journals on the nose, throat and ear during the past year. It is thus a valuable little reference book for the specialist who is unable to keep on file the various special journals which treat these subjects. In writing a paper this book would no doubt prove of value as a quick reference work. However, it is not complete, and we still think that unless a year-book possess this one quality, it will be difficult to get it yearly into the library of physicians. From its preface we gather that this is the first issue of such a book, and no doubt the future editions will show marked improvements. The idea is certainly a good one, and for those physicians who live where a large medical library cannot be consulted, such a work as this will prove of great value. The arrangement in the book is good, and with a more extended experience we believe that the authors will make this a volume of merit. They recognize this by saying in the preface: "However, as an epitome of much of the best literature of the year put into a volume convenient for reference, they are willing to submit it to the medical public, trusting that with experience gained in the preparation of this, they may be able to offer a very much more complete and valuable book for the next issue."

